# A Retrospective Cross-Sectional Study on the Clinicopathologic Features of Essential Hypertension at High Altitude in China

**DOI:** 10.1155/cdr/8834620

**Published:** 2025-11-25

**Authors:** Meiting Gong, Hongyang Zhang, Meiling Li, Qian Chen, Xianglin Ye, Jie Tang, Hao Liu, Haifeng Pei, Feipeng Wei

**Affiliations:** ^1^Department of Cardiology, The General Hospital of Western Theater Command, Chengdu, China; ^2^Department of Clinical Medicine, Southwest Medical University, Luzhou, China; ^3^Physical Examination Center, The General Hospital of Western Theater Command, Southwest Jiaotong University, Chengdu, China; ^4^Department of Vascular Surgery, Xi'an No. 3 Hospital, The Affiliated Hospital of Northwest University, Xi'an, China

**Keywords:** antihypertensive drugs, cardiac structure, coronary heart disease, high-altitude essential hypertension, hyperuricemia

## Abstract

**Purpose:**

The purpose of the study is to analyze the clinicopathological characteristics of essential hypertension (EH) in high-altitude regions of China and provide evidence-based guidance for rational diagnosis and treatment strategies in these areas.

**Methods:**

This cross-sectional retrospective study enrolled two cohorts of EH patients from Qinghai (high altitude, ≥ 2500 m) and Chengdu (low altitude, 500 m) between 2020 and 2022. Participants were stratified based on their residential altitude. Clinical parameters, biochemical markers, cardiac imaging data, and antihypertensive regimens were systematically compared. Statistical analyses were conducted using SPSS software (Version 26.0).

**Results:**

Compared with patients with EH at low altitude, high-altitude EH patients had a higher mean systolic/diastolic blood pressure and a lower percentage of compliance with blood pressure lowering and were more likely to have hyperuricemia (HUA) and abnormally elevated metabolic function (*p* = 0.03). Significant cardiac structural changes occurred in patients with high-altitude EH: increased pulmonary artery internal diameter (*p* < 0.001). In addition, the proportion of angiotensin-converting enzyme inhibitor, angiotensin II receptor blocker, *β*-blocker, and diuretic use was lower in patients with high-altitude EH, while the proportion of SPC combination use was higher; conversely, the proportion of diuretic use was higher in patients with high-altitude EH with comorbid HUA.

**Conclusion:**

EH in high-altitude populations demonstrates distinct clinicopathological manifestations, including right ventricular overload and compensatory left cardiac adaptation. These findings underscore the necessity for altitude-specific clinical guidelines to optimize hypertension management in these regions.

## 1. Introduction

Hypertension is recognized as a global health problem, and the number of hypertensive people in China has reached 245 million by 2022 [1]. Many factors contribute to the development of hypertension, and high-altitude and low-pressure hypoxic environments have been identified as independent risk factors for hypertension [2]. More than half of China's land area is high altitude and mountainous, and more than 70 million people live at altitudes above 1800 m [3]. Previous studies have confirmed that every 100 m of altitude increase can lead to a 2% increase in the prevalence of hypertension [4]. High-altitude hypoxia reduces the body's ability to regulate blood pressure, increases pulmonary artery pressure, and leads to cardiopulmonary remodeling, thus increasing the incidence of stroke and heart failure. Therefore, it is crucial to elucidate the pathogenesis of essential hypertension (EH) at high altitude and to provide rational diagnosis and treatment.

The updated guideline can provide better clinical strategies for hypertension management. The 2023 Guidelines for the Management of Hypertension [5] issued by the European Society of Hypertension (ESH) added “special hypertension phenotypes” to the original classification and staging of hypertension and proposed precise and individualized treatment strategies based on the classification of hypertension. Pharmacological treatment still recommends the use of five major antihypertensive drugs: angiotensin-converting enzyme inhibitor (ACEI), angiotensin II receptor blocker (ARB), *β*-blocker (BB), calcium channel blocker (CCB), and thiazide diuretics, and shows that the use of two antihypertensive drugs as a single-pill combination (SPC) can reduce the daily dosage of medication and increase the therapeutic value of the patients' daily medication intake and improve their treatment adherence and blood pressure control rate. Unfortunately, the existing guidelines for hypertension management do not include a treatment plan for EH at high altitude. Therefore, it is urgent to develop standardized guidelines for the diagnosis and treatment of EH at high altitude.

This is a retrospective cross-sectional study of EH at high altitude and EH at low altitude in China, aiming to provide a useful reference for the development of guidelines for EH at high altitude by exploring the differences in clinical features, structural changes in the heart, and differences in medication choices between EH at high altitude and EH at low altitude.

## 2. Materials and Methods

### 2.1. Study Design and Population

**Figure 1:**
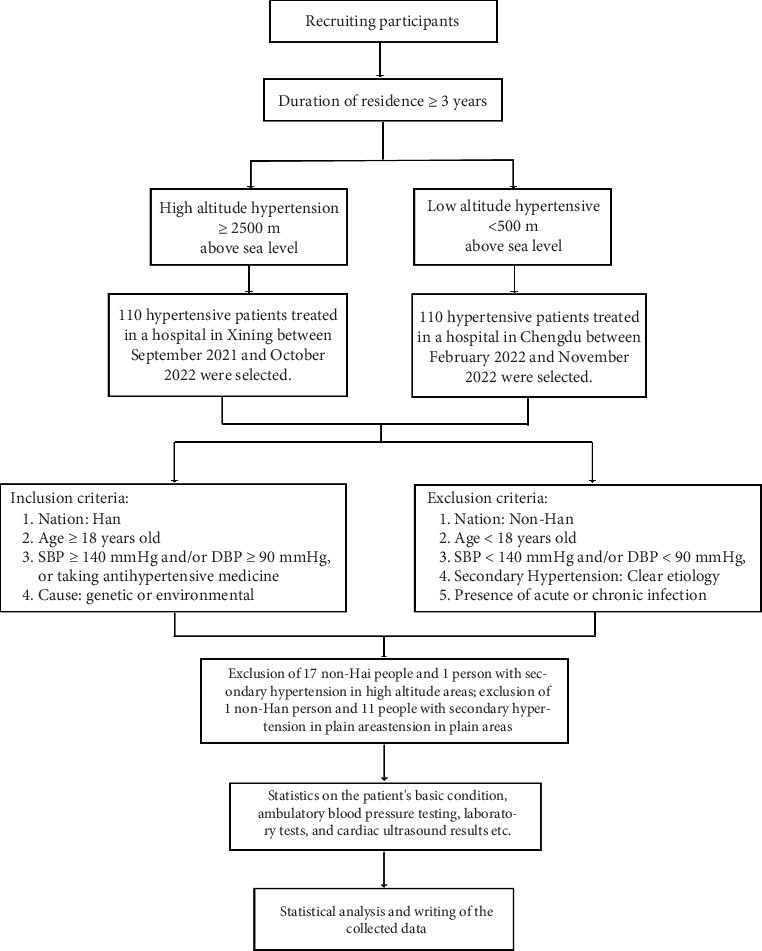
Research object and experimental flowchart.

This was a retrospective cross-sectional study, and previous studies have found that the prevalence of hypertension among adults in low-altitude areas of China is 23.2% [6], while the prevalence of hypertension in high-altitude areas can be as high as 56% [7]. The sample size of 59 patients was calculated using PASS15.0 software according to the significance level *α* of 0.05 and test efficacy 1 − *β* of 0.80. In this study, 110 patients with hypertension at high altitude (altitude ≥ 2500 m) who attended a hospital in Xining, Qinghai, from September 2021 to October 2022 and 110 patients with hypertension at low altitude (altitude ≤ 500 m) who attended a hospital in Chengdu, Sichuan, from February 2022 to November 2022 were collected. To prevent the influence of ethnic adaptation on the results, 17 non-Han nation groups at high altitude and 1 non-Han nation group at low altitude were excluded. Patients were screened and enrolled according to the diagnostic criteria of EH, which are shown in Figure 1, and a total of 1 patient with secondary hypertension at high altitude and 11 patients with secondary hypertension at low altitude were excluded. Finally, a total of 92 patients with EH at high altitude and 98 patients with EH at low altitude were included.

### 2.2. Research Subjects and Experimental Flowchart

Firstly, the consultation information of hypertensive patients in a hospital in Xining, Qinghai, and a hospital in Chengdu, Sichuan, was registered. Patients in both groups had lived at the same altitude for ≥ 3 years, excluding short-term travelers and non-Han populations. They were screened and enrolled according to the diagnostic criteria for adult EH, as shown in Figure 1: (1) nation: Han; (2) age ≥ 18 years; (3) meeting the diagnostic criteria for hypertension, that is, SBP ≥ 140 mmHg and/or DBP ≥ 90 mmHg, or taking antihypertensive medication; and (4) elevated blood pressure caused by genetic or environmental factors. Exclusion criteria are as follows: (1) nation: non-Han; (2) age < 18 years; (3) systolic blood pressure < 140 mmHg and/or diastolic blood pressure < 90 mmHg; (4) belonging to secondary hypertension: the etiology is clear; and (5) the presence of acute or chronic infections. Then, the patients' basic information, ambulatory blood pressure test data, laboratory tests, and cardiac ultrasound results were counted, and finally, the data were organized and analyzed. The study protocol was approved by the Ethics Committee of the Western Theater Command General Hospital (Registration Number 2023EC5-ky001), the Declaration of Helsinki was strictly adhered to, and personal information related to the identity of the patients was concealed.

### 2.3. Definitions

According to the ESH's 2023 European Guidelines for the Management of Arterial Hypertension, hypertension was defined as a systolic blood pressure of 140 mmHg and/or a diastolic blood pressure of 90 mmHg or a history of antihypertensive drug usage. Secondary hypertension was defined as hypertension caused by some identified disease or etiology that could be treated with cause-specific interventions [8, 9]. Smokers were defined as those who had smoked continuously for more than 6 months. Age subgroups were defined as young < 55 years, middle-aged 55–64 years, and older ≥ 65 years, based on the age group in which hypertension occurs at high frequency [10, 11].

### 2.4. Statistical Analysis

The measurement data were tested for normality and chi-square, and an independent samples *t*-test was used when the data obeyed normal distribution, expressed as mean ± SD; the Mann–Whitney *U* test was used when the data did not obey normal distribution, expressed as M (P25, P75). Between-group count data were calculated using the chi-square test with 95% confidence intervals using the online tool VassarStats. After subgroup analysis of patients with EH at high and low altitudes by age (< 55, 55–64, and ≥ 65 years), sex (male and female), and whether or not they had comorbidities (CHD or HUA), measurements were analyzed by between-groups analysis of variance if they met the normal distribution and had a chi-squared variance and by nonparametric *K*-independent samples test if they did not meet the normal distribution or had a chi-squared variance. Differences were considered statistically significant at *p* < 0.05.

## 3. Results

### 3.1. Basic Clinicopathologic Characteristics of Essential Han Chinese Patients With Hypertension at High Altitude in China

The study cohort comprised 92 high-altitude EH patients and 98 low-altitude counterparts. Both Han Chinese groups demonstrated comparable baseline characteristics in age and gender distribution (*p* > 0.05). Compared with patients with EH at low altitude, patients with EH at high altitude were more likely to have comorbid HUA (95% CI: 0.01–0.24, *p* = 0.03) and higher metabolic levels, including TSH (95% CI: −0.96 to −0.04, *p* = 0.32), FT3 (95% CI: −1.06 to −0.39, *p* < 0.001), and FT4 (above the normal range, 95% CI: −14.02 to −12.42, *p* < 0.001). Conversely, they showed lower incidence of coronary heart disease (CHD) (95% CI: 0.03–0.29, *p* = 0.016) and atrial fibrillation (AF) (95% CI: 0.03–0.18, *p* = 0.008) (Table 1). Although there was no statistically significant difference in age and gender between the two groups, subgroup analysis by age and gender revealed that the proportion of smoking was lower in patients ≥ 65 years of age with high altitude EH (*p* = 0.018, Exhibit S1a), especially in men with high-altitude EH (*p* = 0.048, Exhibit S1b). Subgroup analysis by the presence or absence of comorbid CHD revealed that TSH and FT3 levels were higher in high-altitude patients with EH combined with CHD compared with those with EH combined with CHD at low altitude (*p* < 0.001, Exhibit S1c). In contrast, when there was no combined CHD, no difference was seen in TSH and FT3 levels between hypertensive patients in the two regions (*p* > 0.05).

### 3.2. Poor Blood Pressure Control in Han Chinese Patients With EH at High Altitudes

As shown in Table 2, compared with patients with EH at low altitude, patients with EH at high altitude had higher mean systolic/mean diastolic blood pressure (138.74 ± 18.41/79.48 ± 12.89 vs. 129.21 ± 9.8/72.59 ± 8.72 mmHg, *p* < 0.001) and higher mean daytime blood pressure (99.68 ± 12.99 vs. 91.00 ± 9.36 mmHg, *p* < 0.001) and nocturnal mean blood pressure (97.04 ± 16.56 vs. 91.02 ± 7.59 mmHg, *p* < 0.001). Nocturnal blood pressure drop rate SBP and nocturnal blood pressure drop rate DBP were also higher. More importantly, the rate of achieving blood pressure reduction was significantly lower in patients with EH at high altitude than in patients with EH at low altitude (46.7% vs. 81.6%, 95% CI: 0.21–0.47, *p* < 0.001).

### 3.3. Special Cardiac Structural Changes in Han Chinese Patients With EH at High Altitude

Under the high-altitude environment, hypoxia will lead to increased pressure in the pulmonary artery and induce structural changes in the right heart system; at the same time, the human body will compensate for the hypoxic environment by adjusting the structure and function of the left heart system.  Table 3 suggests that, compared with patients with EH at low altitude, patients with EH at high altitude showed an increase in pulmonary artery internal diameter (above normal, 95% CI: −4.00 to −1.00, *p* < 0.001), and patients with EH at high altitude showed a smaller anteroposterior left atrial diameter (95% CI: 2.00–5.00, *p* < 0.001), and EF was higher (95% CI: −8 to −4, *p* < 0.001). Subgroup analyses by the presence or absence of comorbid CHD or HUA revealed (Exhibit S2a and S2b) that when there was non-CHD, compared with patients with EH at low altitude, patients with EH at high altitude had larger pulmonary artery internal diameters (above the range of normal values, *p* = 0.013), smaller anterior and posterior diameters of the left atrium (*p* = 0.01), and higher EF values (*p* < 0.001); when considering whether or not to merge HUA, patients with EH at high altitude had smaller anterior–posterior left atrial diameters (*p* < 0.05) and higher EF (*p* < 0.05), compared with patients with EH at low altitude.

### 3.4. Antihypertensive Drug Regimens Need to Be Adjusted in Han Chinese Patients With EH at High Altitudes

Table 4 suggests that the percentage of medications taken by patients with EH at low altitude is as follows: ACEI 23.5%, ARB 34.7%, BB 52%, diuretics 26.5%, MRA 13.3%, and SPC compound preparation 14.4%, while the percentage of medications taken by patients with EH at high altitude is as follows: ACEI 5.4%, ARB 20.7%, BB 28.3%, diuretics 12%, MRA 10.9%, and SPC compound preparation 26.1%. Statistical analysis revealed that the proportion of patients with EH at high altitude taking ACEI (95% CI: 0.08–0.28, *p* < 0.001), ARB (95% CI: 0.01–0.26, *p* = 0.031), and BB (95% CI: 0.09–0.36, *p* < 0.001) was much lower, while SPC compounding agents took a higher proportion (95% CI: 0.00–0.23, *p* = 0.042). In contrast, CCB, MRA, and diuretic use did not differ between the two groups (*p* > 0.05). Subgroup analysis by gender revealed that the proportion of diuretics taken by male high-altitude EH patients was lower than that of male low-altitude EH patients (17.5% vs. 30%, *p* = 0.001, Exhibit S3a). Subgroup analysis according to the presence or absence of HUA revealed that when there was no comorbidity with HUA, the proportion of diuretics (*p* = 0.012) and BB (*p* = 0.002) was lower in patients with EH at high altitude compared with patients with EH at low altitude; on the contrary, when there was comorbidity with HUA, the difference in the use of the above drugs disappeared between the two groups (*p* > 0.05, Exhibit S3b). At high altitude, a higher proportion of patients with combined HUA took diuretics (*p* = 0.039) compared with those without combined HUA (Exhibit S3b).

## 4. Discussion

In this study, we found that compared with patients with EH at low altitude, (1) a higher proportion of patients with EH at high altitude had combined HUA and metabolic abnormalities, whereas a lower proportion had combined CHD and AF; (2) a higher mean systolic/mean diastolic blood pressure and a lower proportion of patients with EH at high altitude had achieved the standard of blood pressure control; (3) a higher proportion of patients with EH at high altitude had a cardiac overload of the right heart system, and compensation of the left heart system is prone to occur; (4) the proportion of patients with EH at high altitude who take SPC combinations is higher, while the proportion of patients who take ACEI/ARB is lower, and moreover, the proportion of patients with EH at high altitude who take diuretics and BB is higher in patients with comorbid HUA.

Globally, about 1.39 billion people suffer from hypertension, which causes about 10.4 million premature deaths each year [12]. The prevalence of hypertension among people aged 18 years and above in China has reached 27.9% and is on the rise [13]. A retrospective study showed a positive correlation between altitude and prevalence of hypertension [4]. Zhao et al. found that the prevalence of hypertension among Tibetan herders living at an altitude of 4300 m was 55.9%, significantly higher than at lower altitudes [14]. Aryal et al. found that for every 1000 m increase in altitude, there was an increase of 17 mmHg in SBP and 9.5 mmHg in DBP [7]. Wolff et al. suggested that high altitude activates the adrenal and thyroid axes, significantly increasing FT4 levels [15]. At the same time, high-altitude hypoxia induces increased erythropoiesis and purine metabolites and affects renal function so that uric acid excretion is reduced, which leads to an increased incidence of HUA [16]. Consistent with the present study, we also found that Chinese patients with EH at high altitudes are more likely to be combined with increased HUA and abnormalities of thyroid function. Interestingly, Faeh et al. showed that the unique climatic conditions of high altitude were protective against CHD and myocardial infarction (MI), with the risk of CHD and stroke mortality decreasing by 22% and 12% for every 1000-m increase in altitude [17]. Baibas et al. found that Swiss villagers at high altitudes had reduced CHD mortality rates compared to individuals at lower altitudes [18]. We also found that the proportion of combined CHD was lower in patients with EH at high altitudes in China, which may be due in part to the increased physical activity and fitness of high-altitude populations as a result of the rugged geography of high altitude [19], as well as the potential cardiovascular benefits of hypoxia-stimulated inducible factor production and the high levels of vitamin D produced by intense ultraviolet radiation at high altitude, which can reduce the risk of thrombosis and delay the onset of cardiovascular and cerebrovascular disease [20–22]. Nonetheless, mean blood pressure was generally higher, and arterial oxygen saturation was lower in high-altitude residents than in low-altitude residents [23–25]. This phenomenon may occur due to the activation of the sympathetic nervous system (SNS) by the high altitudes hypoxic environment, which affects cardiovascular function and leads to elevated systolic and diastolic blood pressure. In this study, the mean systolic/mean diastolic blood pressure, mean daytime blood pressure, and mean nighttime blood pressure of Chinese patients with EH at high altitudes were higher than those of patients with EH at low altitudes. The attainment rate of EH at high altitudes was significantly lower than that of patients with EH at low altitudes, which further confirmed the significant effect of the hypoxic environment at high altitudes on blood pressure regulation. Therefore, the diagnosis and treatment plan for patients with EH at high altitudes still needs to be standardized and adjusted to improve the prognosis of such patients.

Chronic hypoxia in plateau causes erythrocytosis, increased blood viscosity, and increased pulmonary vascular resistance. At the same time, hypoxia mediates constriction of small pulmonary vessels, increasing pulmonary arterial pressure, leading to right ventricular overload and compensatory left heart remodeling [26, 27]. Prolonged exposure to high altitude induces pulmonary hypertension and vascular remodeling. Ge et al. suggested that high-altitude exposure induces pulmonary hypertension in infants, leading to right ventricular hypertrophy and heart failure [28]. Meanwhile, Rao et al. showed that acute high-altitude exposure may lead to enhanced left ventricular systolic function and increased per-beat output in healthy young men, but their diastolic function is reduced [24]. Osculati et al. also found enhanced left ventricular contractility and left ventricular apical torsion and increased ejection fraction in healthy adults entering high altitudes [29]. Interestingly, Lei et al. found that patients with pulmonary hypertension living at low altitudes had a more pronounced decrease in cardiac function than patients at high altitudes [30]. In this study, compared with Han Chinese hypertensive patients at low altitude, Han Chinese hypertensive patients at high altitude in China had an increased load on the right cardiac system and an increased pulmonary artery internal diameter; at the same time, they had an increased left cardiac system compensation, a decreased anteroposterior diameter of the left atrium, and an increased EF value. Therefore, we believe that the low-pressure, low-oxygen environment at high altitudes can induce pulmonary hypertension, increase proper heart pressure, and lead to the overload of the right cardiac system. The body needs sufficient blood to supply the whole body, and thus, the left cardiac system will be increased to a certain extent to compensate to meet the body's needs. It is well known that a high-purine diet and hypoxic environment in high-altitude populations increase the prevalence of HUA and that uric acid independently predicts the development of hypertension and can exacerbate left ventricular hypertrophy in hypertensive patients [31, 32]. In this study, a higher proportion of Chinese patients with EH at high altitudes had combined HUA compared with patients with EH at low altitudes. When HUA was not combined, compared with patients with EH at low altitude, patients with EH at high altitude in China had more significant pulmonary artery internal diameters, anteroposterior left atrial diameter, and higher EF. When HUA was combined, the difference between the groups decreased. We hypothesized that HUA may adversely affect cardiac structure and function in low-altitude patients with EH. Previous studies have found that HUA can lead to endothelial dysfunction, inflammation, and vasoconstriction by activating oxidative stress, increasing the incidence of hypertension, and damaging target organs [33]. In addition, hypertension is a key risk factor for CHD, and patients with hypertension combined with CHD have increased left ventricular end-diastolic volume, left ventricular end-diastolic wall thickness, and left ventricular diastolic internal diameter, as well as a decrease in their ejection fraction [33]. Our results showed that when CHD was not combined with hypertension, Chinese patients with EH at high altitude had more significant pulmonary artery internal diameters and suitable atrial transverse diameters, smaller anterior and posterior left atrial diameters, and higher ejection fractions than patients with EH at low altitude; on the contrary, when CHD was combined with hypertension, the differences in these indexes between the two groups decreased. We hypothesize that CHD may harm the cardiac structure and function of patients with EH at low altitudes. At the same time, the unique environment of high altitude has a specific protective effect on CHD, which leads to the disappearance of the above differences between patients with EH at high altitude and those at low altitude who have a combination of CHD. Since the clinical studies on patients with EH at high altitudes are relatively few and controversial, the effects of the high altitude environment on cardiac structure and function should be viewed dialectically, and the specific underlying mechanisms need to be further investigated in depth.

Guidelines for managing hypertension and expert consensus provide a systematic, comprehensive, and practical diagnosis and treatment program for hypertension. The regulation of hypertension involves multiple mechanisms, including the SNS, renin–angiotensin–aldosterone system (RAAS), and central and peripheral autonomic cardiovascular regulatory systems. In high-altitude hypoxic environments, the SNS is excited, blood pressure is elevated [34], vascular responses to vasodilators (adenosine and ATP) are weakened, oxidative stress is increased, and intracellular calcium, sodium, and potassium accumulation are reduced [35]. Therefore, the treatment of patients with EH at high altitudes should not wholly follow the treatment strategy for EH at low altitudes but should take into account the effects of the particular environment at high altitudes; however, there are no standardized guidelines for the diagnosis and treatment of EH at high altitude. Only a few studies have shown that the selective *β*1 antagonist nebivolol is more effective in regulating nocturnal blood pressure at high altitudes [36]; the BB carvedilol is effective in controlling EH at high altitudes but decreases arterial hemoglobin oxygen saturation and exercise tolerance [37]. Notably, BBs can act on both *β*1 receptors to target cardiovascular and *β*2 receptors to inhibit pulmonary vasodilation and exacerbate hypoxic pulmonary hypertension. However, current agents are not 100% selective for *β*1 receptors [38], suggesting that drugs with greater selectivity for targeting *β*1 receptors, such as bisoprolol, should be prioritized in drug applications for plateau cardiovascular disease to minimize adverse effects on the pulmonary vasculature. The long-acting ARB can control the blood pressure level of patients with hypertension at an altitude of 3400 m, but its efficacy decreases above 5400 m [39]. Its efficacy is reduced; the combination of telmisartan and nifedipine is effective in controlling blood pressure in ultrahigh-altitude patients acutely exposed to an altitude of 3200 m while improving muscle oxygen utilization and preventing high-altitude pulmonary edema [40–42]. Acetazolamide is commonly used in the prevention of acute mountain sickness and suppresses the elevation of blood pressure and episodes of central sleep apnea that occur with rapid advancement to high altitude [43]. In this study, compared with patients with EH at low altitudes, a lower proportion of Chinese patients with EH at high altitudes took ACEI/ARB analogs, and a higher proportion took SPC compounded preparations, which may be related to the fact that the SPC compounded preparations taken by patients with EH at high altitude contain ACEI/ARB analogs, which also aligns with the guideline emphasizing that SPC compounded preparations are more conducive to improving patients' medication adherence [43, 44]. A comparative analysis of the types of SPC medications the two groups took revealed that patients with EH at low altitudes commonly took Entresto (sacubitril valsartan sodium tablets). In contrast, patients with EH at high altitudes were more likely to take irbesartan hydrochlorothiazide, which may be related to the economic differences between the two regions and the medication habits of physicians (Exhibit S4). Juraschek et al. found that metoprolol significantly increased 12-month serum uric acid levels compared to amlodipine and ramipril [45]. Musini et al. found that thiazide diuretics had a more substantial antihypertensive effect than ACEI/ARB and nonselective BB [46]. However, they decreased potassium and increased uric acid, total cholesterol, and triglyceride levels. Both thiazide diuretics and BB increase serum uric acid levels, adversely affecting hypertensive patients with combined HUA. This study found that the proportion of diuretics and BB was higher in Chinese patients with EH at high altitudes. Subgroup analysis according to the presence or absence of HUA revealed that when there was no combination of HUA, the proportion of diuretics and BB was lower in patients with EH at high altitude compared with those at low altitude, and that when there was a combination of HUA, the difference in the use of the above medications between the two groups disappeared (Exhibit S3b). Moreover, at high altitudes, the proportion of patients taking diuretics and beta-blockers was higher in patients with combined HUA than those without combined HUA (Exhibit S3b). These phenomena are closely related to the medication tendencies of physicians at high altitudes and the lack of knowledge of the side effects of certain medications. Therefore, there is an urgent need to develop rational guidelines for preventing and treating EH at high altitudes to standardize the diagnostic and treatment protocols for patients with EH at high altitudes and improve their prognosis.

In summary, patients with EH at high altitudes have a more pronounced increase in blood pressure. They may have a structural and functional combination of an enlarged right cardiac system with an increased compensatory left cardiac system compared with patients with EH at low altitudes. In treating patients with EH at high altitudes, the high-altitude hypoxic environment alters antihypertensive drugs' metabolism and antihypertensive effects. Therefore, the existing hypertension guidelines cannot be followed thoroughly. When choosing a treatment regimen for patients with EH at high altitudes, clinicians may add pulmonary vasodilator drugs to antihypertensive therapy to reduce pulmonary circulation pressure and improve right heart load. When patients with EH at high altitudes are combined with HUA, the administration rate of thiazide diuretics and BB should be reduced, and ARBs, such as losartan, can be chosen to reduce uric acid production at the same time as lowering blood pressure.

## 5. Strengths and Limitations

This study addresses a critical gap in hypertension research by providing the first comprehensive analysis of EH in a large Han Chinese population residing at high altitudes (≥ 2500 m) in China, an underrepresented region despite its vast territory and substantial population. Through a rigorous comparison between high-altitude and low-altitude cohorts, we integrate clinicopathologic features that encompass population characteristics, comorbidities, blood pressure control, cardiac structural and functional changes, metabolic profiles, and detailed antihypertensive regimens. Our findings reveal distinct pathophysiological and therapeutic demands specific to high altitude, thereby establishing an evidence base for the development of region-specific diagnostic and treatment protocols, which are currently absent in global hypertension guidelines. Of course, this study has some shortcomings, such as a small sample size and various reasons for the difference in baseline blood pressure levels between the two groups. In the future, the sample size can be expanded, and patients with previous hypertension and regular medication can be enrolled to provide guidance information for the rational and standardized use of medication and the formulation of prevention and treatment guidelines for patients with EH in high-altitude areas.

## Figures and Tables

**Table 1:** Basic clinicopathologic characterization of the two groups of Han patients.

**Variable**	**Low altitude (** **n** = 98**)**	**High altitude (** **n** = 92**)**	**Difference and 95% CI**	**Statistics**	**p** **value**
Age, years	67.0 (54.0, 74.0)	70.50 (55.0, 78.75)	−3.00 (−7.00 to 1.00)	−1.356	0.175
< 55	47.00 (36.00, 52.20)	43.50 (31.75, 49.00)	4.00 (−2.00 to 9.00)	−1.399	0.162
55–64	58.56 ± 2.71	58.43 ± 2.93	0.13 (−1.97 to 2.24)	0.130	0.897
≥ 65	73.00 (70.00, 77.00)	77.00 (72.00, 82.00)	−3.00 (−6.00 to −1.00)	2.664	0.008
Female, *n* (%)	38 (38.8)	40 (43.5)	0.05 (−0.09 to 0.18)	0.434	0.510
BMI, kg/m^2^	24.97 (23.03, 27.44)	23.93 (21.58, 26.43)	1.07 (0.02–2.06)	−1.997	0.046
Smoking, *n* (%)	42 (42.9)	27 (29.3)	0.14 (−0.00 to 0.26)	3.745	0.053
Diabetes, *n* (%)	20 (20.4)	24 (26.1)	0.06 (−0.06 to 0.18)	0.860	0.354
CHD, *n* (%)	43 (43.9)	25 (27.2)	0.17 (0.03–0.29)	5.761	0.016
AF, *n* (%)	12 (12.2)	2 (2.2)	0.10 (0.03–0.18)	7.051	0.008
COPD, *n* (%)	17 (17.3)	11 (12.0)	0.05 (−0.05 to 0.15)	1.097	0.295
HTG, *n* (%)	18 (18.4)	17 (18.5)	0.00 (−0.11 to 0.11)	0.00	0.984
History of stroke, *n* (%)	4 (4.1)	4 (4.3)	0.00 (−0.06 to 0.07)	0.008	0.927
HUA, *n* (%)	15 (15.3)	26 (28.3)	0.13 (0.01–0.24)	4.706	0.030
RBC (10^12^/L)	4.58 ± 0.74	5.03 ± 0.75	−0.45 (−0.66 to −0.24)	−4.165	< 0.001
Hb (g/L)	136.65 ± 18.73	151.67 ± 23.88	−15.01 (−21.15 to −8.88)	−4.828	< 0.001
Glu (mmol/L)	5.46 (5.03, 6.65)	5.61 (4.91, 7.40)	−0.12 (−0.48 to 0.25)	−0.719	0.472
HbA1c (mmol/L)	6.10 (5.78, 6.73)	6.21 (5.19, 7.81)	0.13 (−0.37 to 0.70)	−0.440	0.660
TG (mmol/L)	1.33 (0.96, 1.98)	1.59 (1.13, 2.23)	−0.21 (−0.41 to 0.00)	1.940	0.052
HDL-C (mmol/L)	1.29 (1.12, 1.48)	1.27 (0.98, 1.44)	0.04 (−0.07 to 0.14)	−0.638	0.524
LDL-C (mmol/L)	2.81 (2.19, 3.43)	2.52 (1.95, 3.19)	0.23 (−0.04 to 0.51)	−1.651	0.099
TSH (mlU/L)	1.73 (1.10, 2.69)	2.21 (1.45, 3.81)	−0.49 (−0.96 to −0.04)	2.149	0.032
FT3 (pmol/L)	3.05 ± 0.38	3.77 ± 1.29	−0.72 (−1.06 to −0.39)	−4.210	< 0.001
FT4 (pmol/L)	1.24 (1.12, 1.36)	14.51 (1.29, 17.35)	−13.25 (−14.02 to −12.42)	5.642	< 0.001

*Note:* Statistics: *T* or *z* value or chi-square.

Abbreviations: AF, atrial fibrillation; BMI, body mass index; CHD, coronary heart disease; COPD, chronic obstructive pulmonary disease; FT3, free triiodothyronine; FT4, free thyroxine; Glu, glucose; HbA1c, glycosylated hemoglobin; Hb, hemoglobin; HDL-C, high-density lipoprotein cholesterol; HTG, hypertriglyceridemia; HUA, hyperuricemia; LDL-C, low-density lipoprotein cholesterol; RBC, red blood cell; TG, triglyceride; TSH, thyroid-stimulating hormone.

**Table 2:** Analysis of blood pressure control in hypertensive patients from high-altitude and low-altitude areas in China.

**Variable**	**Low altitude (** **n** = 98**)**	**High altitude (** **n** = 92**)**	**Difference and 95% CI**	**Statistics**	**p** **value**
MeanSBP (mmHg)	129.21 ± 9.80	138.74 ± 18.41	−9.53 (−13.79 to −5.25)	−4.410	< 0.001
SD of MeanSBP (mmHg)	12.30 (10.70, 14.5)	14.70 (12.60, 16.50)	−2.20 (−3.10 to −1.30)	4.916	< 0.001
MeanDBP (mmHg)	72.59 ± 8.72	79.48 ± 12.89	−6.89 (−10.06 to −3.72)	−4.285	< 0.001
SD of MeanDBP (mmHg)	8.85 (7.30, 10.03)	10.50 (9.40, 11.88)	−2.00 (−2.60 to −1.40)	6.242	< 0.001
AHR, beats/min	70.00 (64.00, 74.00)	72.50 (65.00, 82.75)	−3.00 (−6.00 to 0.00)	2.123	0.034
SPL daytime > 140 and nighttime > 120 ratio, *N* (%)	47.5 0 (22.00, 65.00)	50.00 (21.50, 81.75)	−5.00 (−15.00 to 4.00)	1.026	0.305
DPL > 90 during the day and > 80 at night ratio, *N* (%)	21.00 (4.75, 51.25)	18.00 (5.25, 34.50)	1.00 (−4.00 to 7.00)	−0.414	0.679
Nocturnal blood pressure drop rate SBP	−0.34 ± 7.74	2.27 ± 8.16	−2.61 (−4.88 to −0.33)	−2.261	0.025
Nocturnal blood pressure drop rate DBP	0.46 ± 8.81	3.19 ± 9.93	−2.73 (−5.42 to −0.05)	−2.010	0.046
Mean daytime BP (mmHg)	91.00 ± 9.39	99.68 ± 12.99	−8.68 (−11.94 to −5.41)	−5.249	< 0.001
Mean nocturnal BP (mmHg)	91.02 ± 7.59	97.04 ± 16.56	−6.02 (−9.75 to −2.28)	−3.185	0.002
Compliance rate, *N* (%)	80 (81.6)	43 (46.7)	0.35 (0.21–0.47)	25.309	< 0.001

*Note:* Statistics: *T* or *z* value or chi-square.

Abbreviations: AHR, average heart rate; BP, blood pressure; DPL, diastolic pressure load; MeanDBP, mean diastolic blood pressure; MeanSBP, mean systolic blood pressure; SD, standard deviation; SPL, systolic pressure load.

**Table 3:** Analysis of differences in cardiac structure and function between patients with PH at high and low altitudes in China.

**Variable**	**Low altitude (** **n** = 98**)**	**High altitude (** **n** = 92**)**	**Difference and 95% CI**	**Statistics**	**p** **value**
PAI (mm)	22.00 (20.00, 22.00)	23.00 (20.00, 27.00)	−2.00 (−3.00 to −1.00)	3.313	< 0.001
RATD (mm)	34.00 (32.00, 37.00)	35.00 (32.00, 39.75)	−1.00 (−3.00 to 0.00)	1.450	0.147
AOD (mm)	32.00 (29.75, 35.00)	32.00 (29.00, 35.75)	0.00 (−1.00 to 1.00)	−0.035	0.972
ALAD (mm)	36.50 (34.00, 41.00)	34.00 (31.00, 38.00)	3.00 (2.00–5.00)	−3.908	< 0.001
LVDD (mm)	44.00 (42.00, 47.00)	46.00 (42.00, 51.00)	−1.00 (−3.00 to 1.00)	1.360	0.174
LVDS (mm)	29.00 (28.00, 32.00)	29.00 (25.00, 32.75)	1.00 (0.00–3.00)	−1.751	0.080
EF (%)	60.00 (58.00, 63.00)	66.00 (61.25, 71.00)	−6.00 (−8.00 to −4.00)	5.633	< 0.001

*Note:* Statistics: *z* value or chi-square.

Abbreviations: ALAD, anteroposterior left atrial diameter; AOD, aortic internal diameter; EF, ejection fraction; LVDD, left ventricular end-diastolic internal diameter; LVDS, left ventricular end-systolic internal diameter; PAI, pulmonary artery inner diameter; RATD, right atrial transverse diameter.

**Table 4:** Analysis of medication use among hypertensive patients in high-altitude and low-altitude areas in China.

**Variable**	**Low altitude (** **n** = 98**)**	**High altitude (** **n** = 92**)**	**Difference and 95% CI**	**Statistics**	**p** **value**
ACEI, *n* (%)	23 (23.5)	5 (5.4)	0.18 (0.08–0.28)	12.283	< 0.001
ARB, *n* (%)	34 (34.7)	19 (20.7)	0.14 (0.01–0.26)	4.652	0.031
CCB, *n* (%)	57 (58.2)	55 (59.8)	0.02 (−0.12 to 0.15)	0.051	0.821
BB, *n* (%)	51 (52.0)	26 (28.3)	0.24 (0.09–0.36)	11.133	< 0.001
Diuretic, *n* (%)	26 (26.5)	11 (12.0)	0.15 (0.03–0.25)	6.427	0.011
MRA, *n* (%)	13 (13.3)	10 (10.9)	0.02 (−0.07 to 0.12)	0.256	0.613
SPC, *n* (%)	14 (14.3)	24 (26.1)	0.12 (0.00–0.23)	4.130	0.042

*Note:* Statistics: chi-square.

Abbreviations: ACEI, angiotensin-converting enzyme inhibitor; ARB, angiotensin receptor blocker; BB, beta-blocker; CCB, calcium channel blocker; MRA, mineralocorticoid receptor antagonist; SPC, single-pill combination.

## Data Availability

The data that support the findings of this study are available on request from corresponding author Haifeng Pei, upon reasonable request.

## Supporting Information

Supporting Information Additional supporting information can be found online in the Supporting Information section. Exhibit S1a: Basic characterization of high-altitude and low-altitude hypertensive patients grouped according to age. Exhibit S1b: Basic characterization of high-altitude and low-altitude hypertensive patients grouped according to gender. Exhibit S1c: Basic characterization of high-altitude and low-altitude hypertensive patients grouped according to the presence or absence of comorbid CHD. Exhibit S2a: Structural and functional analysis of the heart in high-altitude and low-altitude hypertensive patients grouped according to the presence or absence of comorbid CHD. Exhibit S2b: Structural and functional analysis of the heart in high-altitude and low-altitude hypertensive patients grouped according to the presence or absence of comorbid. Exhibit S3a: Analysis of medication use in hypertensive patients from high altitude and low altitude grouped according to gender. Exhibit S3b: Analysis of medication use in high-altitude and low-altitude hypertensive patients grouped according to the presence or absence of comorbid hyperuricemia. Exhibit S4: Analysis of SPC drug use in hypertensive patients in high-altitude and low-altitude regions.
