# Supplementary material for: A Retrospective Cross-Sectional Study on the Clinicopathologic Features of Essential Hypertension at High Altitude in China
Source: Cardiovasc Ther. 2025 Nov 25;2025:8834620. doi: 10.1155/cdr/8834620 (PMC12672070; doi:10.1155/cdr/8834620)
Supplement: Supporting Information — Additional supporting information can be found online in the Supporting Information section. Exhibit S1a: Basic characterization of high-altitude and low-altitude hypertensive patients grouped according to age. Exhibit S1b: Basic characterization of high-altitude and low-altitude hypertensive patients grouped according to gender. Exhibit S1c: Basic characterization of high-altitude and low-altitude hypertensive patients grouped according to the presence or absence of comorbid CHD. Exhibit S2a: Structural and functional analysis of the heart in high-altitude and low-altitude hypertensive patients grouped according to the presence or absence of comorbid CHD. Exhibit S2b: Structural and functional analysis of the heart in high-altitude and low-altitude hypertensive patients grouped according to the presence or absence of comorbid. Exhibit S3a: Analysis of medication use in hypertensive patients from high altitude and low altitude grouped according to gender. Exhibit S3b: Analysis of medication use in high-altitude and low-altitude hypertensive patients grouped according to the presence or absence of comorbid hyperuricemia. Exhibit S4: Analysis of SPC drug use in hypertensive patients in high-altitude and low-altitude regions. [file 8834620.f1.docx]

**Supplemental Materials**

**Exhibit 1a: Basic Characterization of high altitude and low altitude Hypertensive Patients Grouped According to Age**

|  | <55 years old | | |  | 55-64 years old | | |  | ≥65 years old | | | |
| --- | --- | --- | --- | --- | --- | --- | --- | --- | --- | --- | --- | --- |
| Variable | Low altitude (n=25) | High altitude (n=22) | *P*-value |  | Low altitude (n=16) | High altitude (n=14) | *P*-value |  | Low altitude (n=57) | | High altitude (n=56) | *P*-value |
| BMI, kg/m2 | 26.98(24.19, 28.50) | 25.39(24.24, 27.93) | 0.382 |  | 24.44(23.83, 27.52) | 25.89(23.36, 28.13) | 0.790 |  | 24.41±3.02 | 23.19±3.42 | | 0.049 |
| Smoker, n (%) | 11(44.0) | 8(36.4) | 0.595 |  | 7(43.8) | 7(50.0) | 0.732 |  | 24(42.1) | 12(21.4) | | 0.018 |
| CHD, n (%) | 2(8.0) | 1(4.5) | 0.629 |  | 9(56.3) | 2(14.3) | 0.017 |  | 32(56.1) | 22(39.3) | | 0.073 |
| HUA, n (%) | 1(4.0) | 5(22.7) | 0.055 |  | 2(12.5) | 5(35.7) | 0.134 |  | 12(21.1) | 16(28.6) | | 0.355 |
| RBC (10^12/L) | 4.93±0.63 | 5.03±0.59 | 0.587 |  | 4.64(4.31, 4.78) | 5.28(4.96, 5.77) | ＜0.001 |  | 4.43±0.81 | 4.91±0.67 | | ＜0.001 |
| Hb (g/L) | 143.00(127.00, 160.00) | 156.50(143.00, 174.40) | 0.043 |  | 141.81±13.84 | 165.43±29.97 | 0.015 |  | 133.07±18.84 | 147.96±21.73 | | ＜0.001 |
| TG (mmol/L) | 1.45(1.16, 2.21) | 1.90(1.27, 2.57) | 0.258 |  | 1.33±0.62 | 2.11±0.95 | 0.017 |  | 1.32(0.95, 2.03) | 1.42(1.08, 1.76) | | 0.508 |
| HDL-C (mmol/L) | 1.27±0.27 | 1.28±0.37 | 0.950 |  | 1.32±0.22 | 1.59±0.29 | 0.037 |  | 1.34(1.12, 1.45) | 1.23(0.96, 1.39) | | 0.224 |
| LDL-C (mmol/L) | 2.80(2.43, 3.48) | 2.64(1.91, 3.73) | 0.394 |  | 2.74±1.12 | 2.78±0.57 | 0.900 |  | 2.81±1.14 | 2.44±0.79 | | 0.054 |
| TSH (mlU/L) | 1.86(1.35, 2.40) | 1.92(1.60, 2.35) | 0.679 |  | 1.73(1.37, 2.79) | 1.87(0.79, 2.98) | 0.815 |  | 1.59(0.99, 3.09) | 2.63(1.56, 4.76) | | 0.012 |
| FT3 (pmol/L) | 3.28±0.39 | 3.44±1.77 | 0.774 |  | 3.12±0.21 | 3.82±1.74 | 0.214 |  | 2.92±0.36 | 3.85±0.97 | | ＜0.001 |
| FT4 (pmol/L) | 1.26(1.14, 1.45) | 1.59(0.83, 17.47) | 0.542 |  | 1.20(1.17 to 1.36) | 13.67(0.90, 16.27) | 0.186 |  | 1.21(1.11, 1.35) | 14.72(13.11, 18.23) | | ＜0.001 |

**Note:** BMI: Body Mass Index; CHD: coronary heart disease; HUA: hyperuricemia; RBC: Red Blood Cell; Hb: hemoglobin; TG: Triglyceride; HDL-C: high density lipoprotein-cholesterol; LDL: low density lipoprotein-cholesterol; TSH: thyroid stimulating hormone; FT3: freetriiodothyronin; FT4: free thyroxine.

**Exhibit 1b: Basic Characterization of high altitude and low altitude Hypertensive Patients Grouped According to Gender**

|  | Low altitude (n=98) | |  | High altitude (n=92) | | *P*-value ^a^ |  |  |  |
| --- | --- | --- | --- | --- | --- | --- | --- | --- | --- |
| Variable | Male (n=60) | Female (n=38) |  | Male (n=52) | Female (n=40) |  | *P*-value ^b^ | *P*-value ^c^ | *P*-value ^d^ |
| Age, years | 68(54.50, 74.00) | 67.00(53.75, 73.25) |  | 61.50(44.50, 77.00) | 75.00(69.25, 80.50) | 0.830 | 0.001 | 0.372 | 0.003 |
| BMI | 24.90(23.30, 27.44) | 24.97(22.83, 27.61) |  | 24.82(22.19, 26.41) | 22.85(21.33, 26.65) | 0.971 | 0.205 | 0.338 | 0.034 |
| Smoker, n (%) | 41(68.3) | 1(2.6) |  | 26(50.0) | 1(2.5) | ＜0.001 | ＜0.001 | 0.048 | 0.971 |
| CHD, n (%) | 33(55.0) | 10(26.3) |  | 9(17.3) | 16(40.0) | 0.005 | 0.015 | ＜0.001 | 0.200 |
| HUA, n (%) | 13(21.7) | 2(5.3) |  | 16(30.8) | 10(25.0) | 0.028 | 0.542 | 0.273 | 0.016 |
| RBC (10^12/L) | 4.74±0.83 | 4.33±0.47 |  | 5.24±0.77 | 4.77±0.64 | 0.003 | 0.002 | 0.001 | 0.001 |
| Hb (g/L) | 141.55±19.48 | 128.92±14.65 |  | 159.00(149.00, 173.00) | 144.00(133.25, 151.75) | ＜0.001 | ＜0.001 | ＜0.001 | ＜0.001 |
| TG (mmol/L) | 1.33(0.96, 1.97) | 1.34(0.99, 2.14) |  | 1.62(1.13, 2.22) | 1.50(1.20, 2.39) | 0.932 | 0.953 | 0.133 | 0.207 |
| HDL-C (mmol/L) | 1.22(1.07, 1.40) | 1.42(1.30, 1.56) |  | 1.25(0.96, 1.42) | 1.29(1.15, 1.55) | ＜0.001 | 0.560 | 0.723 | 0.137 |
| LDL-C (mmol/L) | 2.86±1.17 | 2.83±0.87 |  | 2.56(2.05, 2.95) | 2.44(1.79, 3.50) | 0.904 | 0.888 | 0.185 | 0.521 |
| TSH | 1.54(1.01, 2.19) | 2.32(1.49, 3.38) |  | 2.09(1.27, 3.81) | 2.42(1.64, 3.75) | 0.008 | 0.485 | 0.043 | 0.491 |
| FT3 | 3.07±0.40 | 3.02±0.36 |  | 3.95±1.44 | 3.57±1.10 | 0.518 | 0.264 | ＜0.001 | 0.013 |
| FT4 | 1.28±0.16 | 1.19±0.19 |  | 15.98(1.59, 19.65) | 13.67(1.17, 15.56) | 0.032 | 0.162 | ＜0.001 | ＜0.001 |

**Note:** a: Comparison between Male and Female groups among patients in low-altitude regions; b: Comparison between Male and Female groups among patients in high-altitude regions; c: Comparative analysis of low-altitude versus high-altitude regions within the Male cohort; d: Comparative analysis of low-altitude versus high-altitude regions within the Female cohort. BMI: Body Mass Index; CHD: coronary heart disease; HUA: hyperuricemia; RBC: Red Blood Cell; Hb: hemoglobin; Glu: glucose; TG: Triglyceride; HDL-C: high density lipoprotein-cholesterol; LDL-C: low density lipoprotein-cholesterol; TSH: thyroid stimulating hormone; FT3: free triiodothyronin; FT4: free thyroxine.

**Exhibit 1c: Basic Characterization of high altitude and low altitude Hypertensive Patients Grouped According to the Presence or Absence of Comorbid Coronary Heart Disease**

|  | Low altitude (n=98) | |  | High altitude (n=92) | | *P*-value ^a^ |  |  |  |
| --- | --- | --- | --- | --- | --- | --- | --- | --- | --- |
| Variable | Non-CHD (n=55) | Comorbid CHD(n=43) |  | Non-CHD(n=67) | Comorbid CHD (n=25) |  | *P*-value ^b^ | *P*-value ^c^ | *P*-value ^d^ |
| Age, years | 59.20±16.02 | 69.60±8.39 |  | 65.00(49.00, 76.00) | 79.00(71.50, 84.50) | ＜0.001 | ＜0.001 | 0.383 | 0.001 |
| Female，n (%) | 28(50.9) | 10(23.3) |  | 24(35.8) | 16(64.0) | 0.005 | 0.015 | 0.094 | ＜0.001 |
| BMI, kg/m2 | 25.71(23.43, 28.05) | 24.15(22.56, 26.15) |  | 24.51±3.56 | 23.43±3.41 | 0.006 | 0.196 | 0.027 | 0.344 |
| Smoker, n (%) | 19(34.5) | 23(53.5) |  | 23(34.3) | 4(16.0) | 0.060 | 0.086 | 0.980 | 0.002 |
| COPD, n (%) | 8(14.5) | 9(20.9) |  | 9(13.4) | 2(8.0) | 0.407 | 0.475 | 0.860 | 0.163 |
| AF, n (%) | 7(12.7) | 5(11.6) |  | 0(0.0) | 2(8.0) | 0.869 | 0.019 | 0.003 | 0.635 |
| HUA, n (%) | 7(12.7) | 8(18.6) |  | 18(26.9) | 8(32.0) | 0.423 | 0.627 | 0.054 | 0.209 |
| RBC (10^12/L) | 4.67±0.65 | 4.48±0.83 |  | 5.07±0.75 | 4.93±0.73 | 0.213 | 0.417 | 0.002 | 0.028 |
| Hb (g/L) | 138.00±16.66 | 134.93±21.18 |  | 152.00(137.75, 166.25) | 145.00(139.50, 157.00) | 0.424 | 0.278 | ＜0.001 | 0.018 |
| Glu (mmol/L) | 5.36(4.92, 6.32) | 5.71(5.08, 7.90) |  | 5.58(4.79, 7.40) | 6.12(5.23, 8.05) | 0.132 | 0.241 | 0.418 | 0.620 |
| HbA1c (mmol/L) | 6.00(5.70, 6.65) | 6.20(5.80, 6.95) |  | 6.14±1.18 | 6.31±0.77 | 0.170 | 0.788 | 0.830 | 1.000 |
| TG (mmol/L) | 1.33(0.99, 2.10) | 1.34(0.90, 1.95) |  | 1.61(1.16, 2.25) | 1.39(1.12, 2.13) | 0.655 | 0.243 | 0.088 | 0.530 |
| HDL-C (mmol/L) | 1.31(1.17, 1.52) | 1.28(1.10, 1.46) |  | 1.21(0.96, 1.39) | 1.49(1.25, 1.90) | 0.185 | 0.008 | 0.033 | 0.012 |
| LDL-C (mmol/L) | 2.95±0.86 | 2.72±1.28 |  | 2.52(1.82, 3.11) | 2.61(2.11, 3.26) | 0.315 | 0.579 | 0.016 | 0.813 |
| TSH (mlU/L) | 1.98(1.39, 3.29) | 1.42(0.96, 2.32) |  | 2.04(1.34, 3.45) | 2.66(1.84, 4.90) | 0.014 | 0.093 | 0.953 | 0.002 |
| FT3 (pmol/L) | 3.11±0.39 | 2.96±0.36 |  | 3.64±1.36 | 4.01±1.12 | 0.048 | 0.289 | 0.025 | ＜0.001 |
| FT4 (pmol/L) | 1.25±0.19 | 1.24±0.16 |  | 14.46(1.01, 17.47) | 14.60(13.59, 18.05) | 0.845 | 0.268 | 0.002 | ＜0.001 |

**Note:** a: Comparison between non-CHD and CHD groups among patients in low-altitude regions; b: Comparison between non-CHD and CHD groups among patients in high-altitude regions; c: Comparative analysis of low-altitude versus high-altitude regions within the non-CHD cohort; d: Comparative analysis of low-altitude versus high-altitude regions within the CHD cohort. BMI: Body Mass Index; CHD: coronary heart disease; AF: atrial fibrillation; COPD: chronic obstructive pulmonary disease; HUA: hyperuricemia; RBC: Red Blood Cell; Hb: hemoglobin; Glu: glucose; HbA1c: glycosylated hemoglobin; TG: Triglyceride; HDL-C: high density lipoprotein-cholesterol; LDL-C: low density lipoprotein-cholesterol; TSH: thyroid stimulating hormone; FT3: freetriiodothyronin; FT4: free thyroxine.

**Exhibit 2a: Structural and Functional Analysis of the Heart in high altitude and low altitude Hypertensive Patients Grouped According to the Presence or Absence of Comorbid Coronary Heart Disease**

|  | Low altitude (n=98) | |  | High altitude (n=92) | | *P*-value ^a^ |  |  |  |
| --- | --- | --- | --- | --- | --- | --- | --- | --- | --- |
| Variable | Non-CHD (n=55) | Comorbid CHD (n=43) |  | Non-CHD (n=67) | Comorbid CHD (n=25) |  | *P*-value ^b^ | *P*-value ^c^ | *P*-value ^d^ |
| PAI (mm) | 22.00(20.00, 22.00) | 22.00(20.00, 22.00) |  | 23.00(20.00, 28.00) | 24.00(21.00, 26.50) | 0.426 | 0.758 | 0.013 | 0.019 |
| RATD (mm) | 34.31±4.61 | 34.78±2.76 |  | 35.00(32.00, 40.00) | 36.00(32.50, 38.50) | 0.637 | 0.748 | 0.107 | 0.505 |
| AOD (mm) | 32.00±3.72 | 32.44±4.78 |  | 31.84±4.27 | 33.72±4.09 | 0.673 | 0.060 | 0.842 | 0.309 |
| ALAD (mm) | 36.00(34.00, 40.00) | 37.00(35.00, 42.00) |  | 33.00(30.00, 38.00) | 36.00(32.00, 38.50) | 0.300 | 0.103 | 0.001 | 0.038 |
| LVDD (mm) | 44.50(43.00, 49.00) | 44.00(41.00, 46.00) |  | 46.66±6.79 | 46.72±7.47 | 0.070 | 0.969 | 0.718 | 0.078 |
| LVDS (mm) | 30.00(28.00, 34.00) | 29.00(27.00, 31.00) |  | 28.00(25.00, 32.00) | 30.00(26.50, 33.50) | 0.044 | 0.218 | 0.010 | 0.466 |
| EF(%) | 60.00(57.75, 63.00) | 60.50(59.25, 63.00) |  | 67.00(62.00, 72.00) | 63.00(58.00, 67.00) | 0.318 | 0.014 | ＜0.001 | 0.244 |

**Note:** a: Comparison between non-CHD and CHD groups among patients in low-altitude regions; b: Comparison between non-CHD and CHD groups among patients in high-altitude regions; c: Comparative analysis of low-altitude versus high-altitude regions within the non-CHD cohort; d: Comparative analysis of low-altitude versus high-altitude regions within the CHD cohort. CHD: coronary heart disease; PAI: Pulmonary Artery Inner diameter; RATD: right atrial transverse diameter; AOD: aortic diameter ; ALAD: anteroposterior left atrial diameter; LVDD: Left ventricular end-diastolic internal diameter; LVDS: Left ventricular end-systolic internal diameter; EF: ejection fraction.

**Exhibit 2b: Structural and Functional Analysis of the Heart in high altitude and low altitude Hypertensive Patients Grouped According to the Presence or Absence of Comorbid Hyperuricemia**

|  | Low altitude (n=98) | |  | High altitude (n=92) | | *P*-value ^a^ |  |  |  |
| --- | --- | --- | --- | --- | --- | --- | --- | --- | --- |
| Variable | Non-HUA (n=83) | Comorbid HUA (n=15) |  | Non- HUA (n=66) | Comorbid HUA (n=26) |  | *P*-value ^b^ | *P*-value ^c^ | *P*-value ^d^ |
| PAI (mm) | 21.00(20.00, 22.00) | 22.00(22.00, 25.00) |  | 23.00(20.00, 28.00) | 22.50(20.00, 26.00) | 0.024 | 0.670 | ＜0.001 | 0.387 |
| RATD (mm) | 34.00(31.75, 36.00) | 36.50(33.25, 38.00) |  | 35.00(32.00, 38.00) | 35.00(33.00, 40.50) | 0.052 | 0.434 | 0.126 | 1.000 |
| AOD (mm) | 31.67±4.14 | 34.50±3.56 |  | 32.41±4.23 | 32.19±4.51 | 0.032 | 0.828 | 0.336 | 0.128 |
| ALAD (mm) | 36.00(34.00, 41.00) | 37.00(36.00, 41.00) |  | 33.50(31.00, 37.25) | 35.00(30.75, 39.00) | 0.159 | 0.546 | ＜0.001 | 0.043 |
| LVDD (mm) | 44.00(41.00, 46.25) | 47.00(44.00, 51.00) |  | 46.52±6.53 | 47.08±8.01 | 0.035 | 0.729 | 0.109 | 0.755 |
| LVDS (mm) | 29.00(28.00, 31.25) | 31.00(29.00, 33.75) |  | 29.00(25.00, 32.25) | 29.00(27.00, 33.25) | 0.046 | 0.362 | 0.119 | 0.155 |
| EF(%) | 60.00(58.00, 63.00) | 60.50(47.50, 63.00) |  | 66.00(62.00, 72.00) | 64.50(59.00, 69.25) | 0.495 | 0.229 | ＜0.001 | 0.031 |

**Note:** a: Comparison between non-HUA and HUA groups among patients in low-altitude regions; b: Comparison between non-HUA and HUA groups among patients in high-altitude regions; c: Comparative analysis of low-altitude versus high-altitude regions within the non-HUA cohort; d: Comparative analysis of low-altitude versus high-altitude regions within the HUA cohort. HUA: hyperuricemia; PAI: Pulmonary Artery Inner diameter; RATD: right atrial transverse diameter; AOD: aortic diameter ; ALAD: anteroposterior left atrial diameter; LVDD: Left ventricular end-diastolic internal diameter; LVDS: Left ventricular end-systolic internal diameter; EF: ejection fraction.

**Exhibit 3a: Analysis of medication use in hypertensive patients from high altitude and low altitude grouped according to gender**

|  | Low altitude (n=98) | |  | High altitude (n=92) | | *P*-value ^a^ |  |  |  |
| --- | --- | --- | --- | --- | --- | --- | --- | --- | --- |
| Variable | Male (n=60) | Female (n=38) |  | Male (n=52) | Female (n=40) |  | *P*-value ^b^ | *P*-value ^c^ | *P*-value ^d^ |
| ACEI, n(%) | 14(23.3) | 9(23.7) |  | 1(1.9) | 4(10.0) | 0.968 | 0.09 | ＜0.001 | 0.105 |
| ARB, n(%) | 19(31.7) | 15(39.5) |  | 12(23.1) | 7(17.5) | 0.429 | 0.512 | 0.311 | 0.031 |
| CCB, n(%) | 36(60.0) | 21(55.3) |  | 27(51.9) | 28(70.0) | 0.643 | 0.080 | 0.390 | 0.178 |
| BB, n(%) | 32(53.3) | 19(50.0) |  | 14(26.9) | 12(30.0) | 0.748 | 0.745 | 0.005 | 0.071 |
| Diuretic, n(%) | 18(30.0) | 8(21.1) |  | 4(7.7) | 7(17.5) | 0.328 | 0.151 | 0.003 | 0.691 |
| MRA, n(%) | 9(15.0) | 4(10.5) |  | 4(7.7) | 6(15.0) | 0.525 | 0.264 | 0.229 | 0.555 |
| SPC, n(%) | 10(16.7) | 4(10.5) |  | 16(30.8) | 8(20.0) | 0.397 | 0.244 | 0.078 | 0.246 |

**Note:** a: Comparison between Male and Female groups among patients in low-altitude regions; b: Comparison between Male and Female groups among patients in high-altitude regions; c: Comparative analysis of low-altitude versus high-altitude regions within the Male cohort; d: Comparative analysis of low-altitude versus high-altitude regions within the Female cohort. ACEI: Angiotensin converting enzyme inhibitors; ARB: angiotensin receptor blocker; CCB: Calcium channel blockers; MRA: mineralocorticoid receptor antagonist; BB: Beta blockers; SPC: Single-Pill Combination.

**Exhibit 3b: Analysis of medication use in high altitude and low altitude hypertensive patients grouped according to the presence or absence of comorbid hyperuricemia**

|  | Low altitude (n=98) | |  | High altitude (n=92) | | *P*-value ^a^ |  |  |  |
| --- | --- | --- | --- | --- | --- | --- | --- | --- | --- |
| Variable | Non-HUA (n=83) | Comorbid HUA (n=15) |  | Non- HUA (n=66) | Comorbid HUA (n=26) |  | *P*-value ^b^ | *P*-value ^c^ | *P*-value ^d^ |
| ACEI, n(%) | 21(25.3) | 2(13.3) |  | 3(4.5) | 2(7.7) | 0.314 | 0.549 | ＜0.001 | 0.558 |
| ARB, n(%) | 28(33.7) | 6(40.0) |  | 14(21.2) | 5(19.2) | 0.639 | 0.833 | 0.091 | 0.148 |
| CCB, n(%) | 45(54.2) | 12(80.0) |  | 39(59.1) | 16(61.5) | 0.062 | 0.829 | 0.551 | 0.221 |
| BB, n(%) | 41(49.4) | 10(66.7) |  | 16(24.2) | 10(38.5) | 0.218 | 0.173 | 0.002 | 0.082 |
| Diuretic, n(%) | 19(22.9) | 7(46.7) |  | 5(7.6) | 6(23.1) | 0.055 | 0.039 | 0.012 | 0.118 |
| MRA, n(%) | 11(13.3) | 2(13.3) |  | 6(9.1) | 4(15.4) | 0.993 | 0.383 | 0.427 | 0.858 |
| SPC, n(%) | 11(13.3) | 3(20.0) |  | 19(28.8) | 5(19.2) | 0.492 | 0.347 | 0.019 | 0.952 |

**Note:** a: Comparison between non-HUA and HUA groups among patients in low-altitude regions; b: Comparison between non-HUA and HUA groups among patients in high-altitude regions; c: Comparative analysis of low-altitude versus high-altitude regions within the non-HUA cohort; d: Comparative analysis of low-altitude versus high-altitude regions within the HUA cohort. ACEI: angiotensin-converting enzyme inhibitor; ARB: angiotensin receptor blocker; CCB: calcium channel blocker; MRA: mineralocorticoid receptor antagonist; SPC: single tablet combination ACEI: angiotensin-converting enzyme inhibitor; ARB: angiotensin receptor blocker; CCB: calcium channel blocker; MRA: mineralocorticoid receptor antagonist; SPC: single tablet combination. Note: ACEI: Angiotensin converting enzyme inhibitors; ARB: angiotensin receptor blocker; CCB: Calcium channel blockers; MRA: mineralocorticoid receptor antagonist; BB: Beta blockers; SPC: Single-Pill Combination.

**Exhibit 4: Analysis of SPC Drug Use in Hypertensive Patients in high altitude and low altitude Regions**

| Variable | Low altitude (n =14 ) | High altitude (n =24) |
| --- | --- | --- |
| Entresto, N (%) | 14(100.0) | 2(8.3) |
| Irbesartan and Hydrochlorothiazide Tablets, N (%) | 0(0.0) | 16(66.7) |
| Valsartan and Amlodipine Tablets, N (%) | 0(0.0) | 3(12.5) |
| Compound Hypotensive Tablets, N (%) | 0(0.0) | 2(8.3) |
| Compound Bendazol and Hydrochlorothiazide Capsules，N (%) | 0(0.0) | 1(4.2) |
